# Sulfate reducing bacteria induce α-synuclein in intestinal and neuronal cells and tissues and inhibit tyrosine hydroxylase in neuronal cells

**DOI:** 10.3389/fnins.2025.1672793

**Published:** 2025-12-18

**Authors:** Sudha B. Singh, Arianne J. Capacio, Cody A. Braun, Amanda Carroll-Portillo, Sephira Ryman, Henry C. Lin

**Affiliations:** 1 Biomedical Research Institute of New Mexico, Albuquerque, NM, United States; 2 Division of Gastroenterology and Hepatology, Department of Internal Medicine, University of New Mexico, Albuquerque, NM, United States; 3 Department of Translational Neuroscience, The Mind Research Network, Albuquerque, NM, United States; 4 Nene and Jamie Koch Comprehensive Movement Disorder Center, Department of Neurology, University of New Mexico, Albuquerque, NM, United States; 5 Medicine Service, New Mexico VA Health Care System, Albuquerque, NM, United States

**Keywords:** Parkinson’s disease, α-synuclein (α-syn), sulfate reducing bacteria, *Desulfovibrio vulgaris* (DSV), tyrosine hydroxylase

## Abstract

**Introduction:**

Parkinson’s disease (PD), a synucleopathy characterized by the presence of α-synuclein (α-syn) aggregates in the brain, is thought to originate in the intestine. *Desulfovibrio*, resident gut sulfate reducing bacteria (SRB), usually found in very low numbers in a healthy gut, are found in higher numbers in PD. In a recent study, a separate group demonstrated that *Desulfovibrio* isolated from both PD patients and healthy subjects were fed to *C. elegans* and were found to increase α-syn aggregates in the head region of the worms. How these bacteria induce α-syn aggregates in the brain through the gut remain unknown. We tested whether *Desulfovibrio* induced α-syn aggregates in the intestinal cells and triggered α-syn secretion from these cells into the growth medium and whether this growth medium (devoid of bacteria) further induced α-syn aggregates in neurons. We also tested whether *Desulfovibrio* directly induced α-syn aggregates in neuronal cells and inhibited protein expression of tyrosine hydroxylase (TH), a key dopamine-producing enzyme. We also tested whether *Desulfovibrio* increased α-syn levels in intestine, plasma, and in brain in mice.

**Methods:**

Enteroendocrine STC-1 and neuronal SH-Sy5y cells were infected with *Desulfovibriovulgaris* (DSV). We measured α-syn aggregation by immunofluorescence. α-syn levels in cell culture supernatant (sup), tissues, and plasma were measured by enzyme-linked immunosorbent assay (ELISA). Protein expression of TH and α-syn was analyzed by Western blot.

**Results:**

We found that DSV increased the number of STC-1cells with α-syn aggregates and also induced α-syn expression in these cells. Sup from DSV-infected STC-1 had higher α-syn levels compared to control uninfected sup and could induce α-syn aggregates in SH-Sy5y. DSV also directly induced a syn aggregates and inhibited TH in SH-Sy5y. DSV-gavaged mice had higher levels of α-syn in the duodenum, plasma, and in brain and also showed a decreasing trend in TH expression in the brain.

**Conclusion:**

Thus, our findings provide a gut-to-brain link tied to the ability of SRB in the gut-to increase expression, aggregation and spread of α-syn and decrease of TH in PD.

## 1 Introduction

Parkinson’s disease (PD) is a neurodegenerative condition thought to be driven by the aggregation of misfolded protein α-synuclein (α-syn) forming the Lewy bodies in the brain that can be detected at autopsy in case of familial PD with α-synucelin SNCA mutation (Polymeropoulos et al., 1997) and sporadic PD (Spillantini et al., 1997; Baba et al., 1998). Braak hypothesis suggested that PD may originate in the peripheral nervous sytem such as in the gastrointestinal tract and not the brain itself (Braak et al., 2003) Evidence suggests that misfolding and aggregation of α-syn may originate in the gut and spread to the central nervous system (Ryman et al., 2023; Menozzi et al., 2021). Specifically, gut-related prodromal symptoms are often found many years in advance before the onset of motor dysfunction and other pathophysiological features in PD patients (Kim and Sung, 2015). A proof-of-concept study demonstrated a central role of gut-brain axis in the development of PD showed that injecting α-syn fibrils (misfolded and aggregated a syn) into the gut caused α-syn pathology in enteric nervous system (ENS) which further spread to the brain regions in mice (Kim et al., 2019). In mice that underwent vagotomy, aggregates were not observed in the brain implying that α-syn aggregates were transported to the brain from the intestine via the vagus nerve. Presence of α-syn accumulation in the vagus nerve as well as in the ENS have also been reported (Fasano et al., 2015; Beach et al., 2010). How the journey of α-syn begins in the gut is not known.

Gut microbial dysbiosis is observed in PD patients (Scheperjans et al., 2015; Huang et al., 2021, 2023). Sulfate Reducing Bacteria (SRB), specifically strains of *Desulfovibrio*, are rare microbial residents of the gut that are reported to be abnormally high in number in the stool of patients with PD (Murros et al., 2021; Nie et al., 2023). Mounting evidence suggest a contributory role of SRB in the development of diseases (Singh et al., 2023). Our previous studies have reported that *Desulfovibrio vulgaris* (DSV) induced proinflammatory pathways in immune cells as well as increased intestinal permeability in intestinal epithelial cells suggesting that *Desulfovibrio* may contribute to the inflammation and leaky gut in many diseases including PD (Mou et al., 2022; Bartl et al., 2022). In a recent study, *Desulfovibrio* species isolated from stool of PD patients and their healthy spouses were found to increase α-syn aggregates in the head region when fed to *C. elegans* (Huynh et al., 2023) suggesting that *Desulfovibrio* may be a causative agent in the onset of pathological changes observed in PD. However, how gut and neuronal cells are linked by these bacteria are not known. Dopaminergic neurons appear to be particularly susceptible to degeneration in PD. Tyrosine hydroxylase (TH) is a key enzyme involved in dopamine synthesis as it catalyzes the conversion of L-tyrosine to L-3,4-dihydroxyphenylalanine (L-DOPA) in the biosynthesis of dopamine. PD patients have decreased TH in their brain (Rausch et al., 2022; Nakashima et al., 2009) and α-syn can inhibit TH function (Perez et al., 2002). In this study, we tested the hypothesis that *Desulfovibrio* may induce α-syn expression and aggregation in intestinal cells and that aggregation-inducing signals from DSV-infected intestinal cells could be relayed to neuronal cells to cause further α-syn aggregation in these cells, even in the absence of their exposure to DSV. We also tested whether DSV could directly induce a syn aggregation in neuronal cells and inhibit tyrosine hydroxylase (TH) protein expression. We further confirmed our findings in animal models where mice were orally gavaged with DSV for 24 h and α-syn levels were measured in the intestinal and brain lysates as well as in plasma and TH expression was also analyzed in the brain lysates.

## 2 Materials and methods

### 2.1 Cell culture

Mouse small intestinal enteroendocrine cells (STC-1) and Human neuronal (SH-Sy5y) cells were purchased from ATCC (Manassas, VA). STC-1 cells were grown in DMEM containing 10% FBS (ThermoFisher Scientific, Waltham, MA)_._ SH-Sy5y (Sy5y) cells were grown in 1:1 mixture of Eagles minimum essential medium and F12 medium with 10% FBS. Cell cultures were incubated at 37 °C in a humidified incubator with 5% CO_2._ Cells were plated at a density of 1 × 10^6^ one day before infection with DSV.

### 2.2 *Desulfovibrio vulgaris* (DSV) growth

*Desulfovibrio vulgaris* Hildenborough (ATCC 29579, Manassas, VA) was grown anaerobically in Hungate tubes using Postgate’s organic liquid medium with the following composition: 10.56 mM Na_2_SO_4_, 13.29 mM MgSO_4_, 4.12 mM L-Cysteine, 0.4% sodium lactate (60% syrup), 0.4% yeast extract, and 0.5% tryptone. Cultures were grown for 24 h in 5 mL aliquots at 37 °C. Bacteria were counted using Quantom Tx cell counter (Logos Biosystems, South Korea). *D. piger* (ATCC 29098) was also grown in hungate tubes in anaerobic conditions in Postgate’s medium, similar to DSV. On the day of infection, DSV was centrifuged at 6000 rpm for 5 min and bacteria in the pellet were resuspended in phosphate buffered saline (PBS). Cells were infected with various multiplicity of infection (MOI) of DSV for 24 h. Control cells were treated with PBS alone.

### 2.3 Animals

Sixteen five-week-old female C57Bl/6 (15–20 g) mice were purchased from Charles River Laboratories (Wilmington, DE, USA). Study protocol was approved by the Institutional Animal Care and Use Committee at the New Mexico VA Health Care System following guidelines provided by the Guide for the Care and Use of Animals of the National Research Council. Upon arrival, animals were housed in polypropylene cages in a group of 4 mice per cage. Animals were subjected to a 12-h light/dark cycle and kept on a standard rodent diet (Teklad Global Rodent Diets, 2,920X). After 1 week of acclimatization, mice were randomly divided into two groups, Control and DSV. Mice in DSV group were orally gavaged with 100 μL of DSV (109 bacteria) resuspended in lactulose solution (33 mg/mL) from Cumberland Pharmaceuticals (Kristalose; Nashville, TN, USA) as described previously (Singh et al., 2024; Coffman et al., 2024). Lactulose was used as a fermentable substrate for hydrogen-producing gut bacteria, where hydrogen serves as a substrate to promote H2S production by DSV. Control mice were gavaged with 100 μL lactulose only in PBS. After 24 h, mice were euthanized by CO2 asphyxiation. Briefly, mice were euthanized by CO2 hypoxia using 30 to 70% of the chamber or cage volume/min. Upon confirmation of no heartbeat, a secondary measure of cervical dislocation was carried out. Intestinal and brain tissues were collected in RNA/DNA Shield (Zymo Research, Irvine, CA, USA). Blood was collected via cardiac puncture. For brain, mid-brain area was identified (site of substantia nigra with dopaminergic neurons) and collected after observing a technique described previously (Salvatore et al., 2012). This resulted in isolating a section potentially enriched in dopaminergic neurons. A single gavage with incubation period of 24 h was selected to potentially capture the immediate gut response of synuclein in the presence of DSV.

### 2.4 Enzyme-linked immunosorbent assay (ELISA)

α-synuclein level was measured in the filtered STC-1 culture supernatant collected from uninfected or DSV-infected cells using a mouse α-syn ELISA kit from Abcam (ab282865) using manufacturer’s protocol. Same kit was used for measuring α-syn in plasma, intestinal (5 mg/sample) and brain lysates (5 mg/sample) from control or DSV- gavaged animals.

### 2.5 Western blot

Cells were lysed in Lysis buffer in the presence of protease and phosphatase inhibitors as previously described (Singh et al., 2022). Briefly, cell lysis was carried out for 20 min on ice. Lysates were centrifuged at 12,000 rpm for 5 min at 4 °C and supernatants collected and protein concentration determined with Bradford reagent (ThermoFisher Scientific). Tissues were homogenized in RIPA buffer (Cell Signaling technology) containing protease and phosphatase inhibitors using a Tissue Lyser II (Qiagen) and incubated on ice for 30 min following which the lysates were centrifuged and supernatants containing the protein lysates were collected. Fifty microgram of protein samples were run on SDS-PAGE (4–20% tris-glycine) and transferred to nitrocellulose membranes. Membranes were blocked in 5% milk in PBS-T (0.1%Tween 20) for 30 min followed by overnight incubation in antibodies against Actin (Cell Signaling Technology: 4970) and α-synuclein (Cell Signaling Technology: 2642), and tyrosine hydroxylase (Cell Signaling Technology: 58844). Antibodies were diluted as recommended by the manufacturer. Blots were incubated with secondary antibodies (Cell Signaling Technology: 7074) at room temperature for 1 h and developed using enhanced Chemiluminescence HRP signal (ThermoFisher Scientific).

### 2.6 Immunofluorescence

Cells were grown on coverslips in 6-well plates. After treatment, cells were fixed with 4% paraformaldehyde for 15 min and washed with PBS 3 times for 5 min. Cells were incubated in a blocking solution consisting of 5% FBS and 0.3%Triton-X 100 for 1 h. This was followed by overnight incubation with anti- α -syn aggregate antibody (Abcam: ab209538) at 4 °C. Cells were then washed with PBS followed by incubation with Alexa fluor 488 -labeled secondary anti-rabbit antibody (ThermoFisher Scientific:) for 2 h at room temperature. Imaging was done with Olympus Fluoview FV1200 confocal microscope. Aggerates were identified manually in Z- projected images using a defined size threshold (>1 mm in diameter). Cells were scored positive if they contained one or more aggregates exceeding this size. Counting was performed consistently across all controls and treatment groups, and atleast ~150–225 cells per condition per experiment were analyzed across minimum of three independent times. The percentage of positive cells was used for statistical comparisons.

### 2.7 Statistical analysis

All graphs were generated using GraphPad Prism 9 (GraphPad Software, San Diego, CA). Our Data represents means+/= SEM from three independent experiment, each performed with three biological replicates pooled per condition per experiment. Fold change differences were represented as values normalized to control (set as 1). Students *t*-test was used for statistical analysis for all the figures. *p* < 0.05 were considered statistically significant.

## 3 Results

### 3.1 *Desulfovibrio vulgaris* (DSV) induced increase in α-syn aggregation and protein expression in intestinal enteroendocrine STC-1 cells

**Figure 1:**
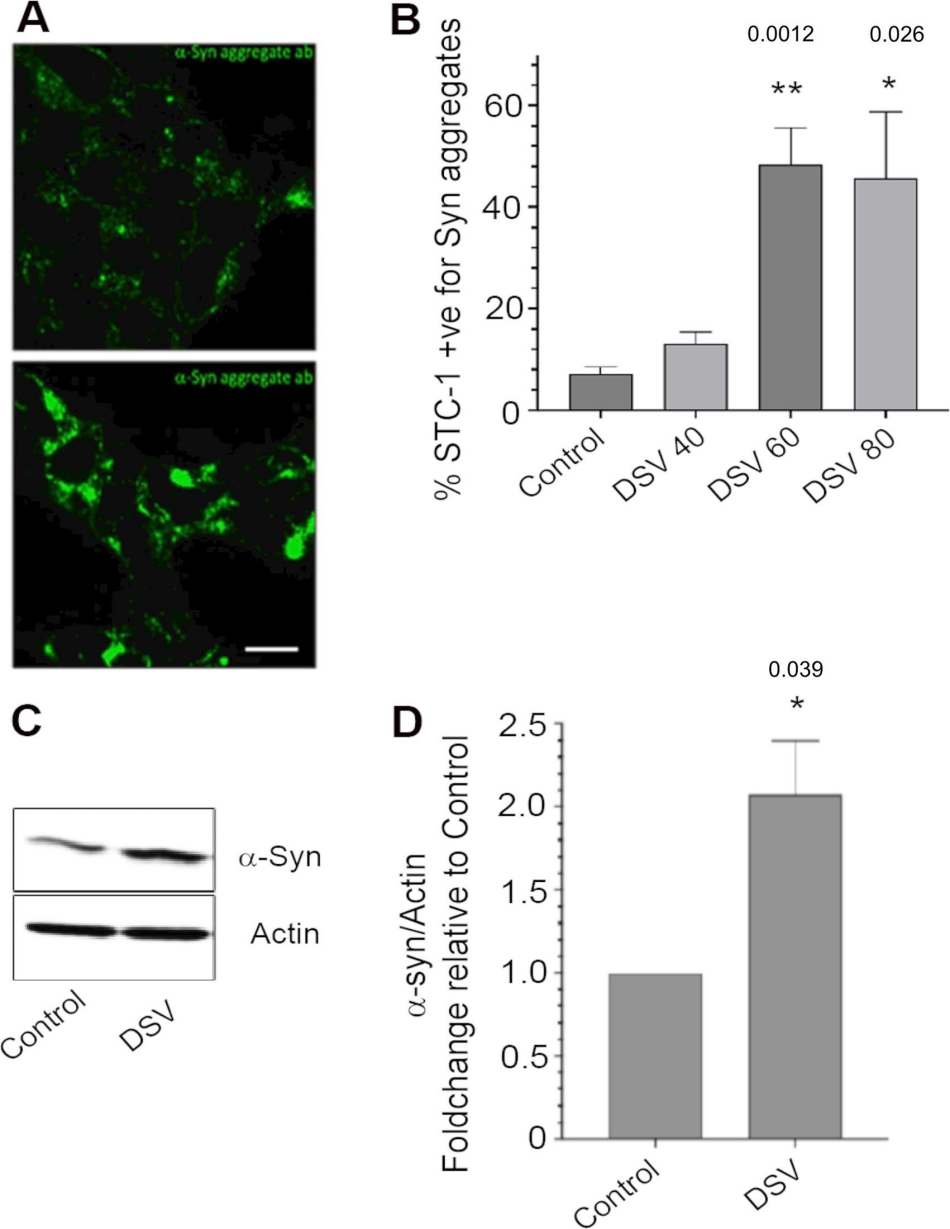
DSV induces increase in number of cells with α-synuclein aggregates and synuclein expression in intestinal enteroendocrine STC-1 cells **(A)**. Cells were infected for 24 h. with various DSV MOIs. Cells were then fixed and processed for immunofluorescence using an antibody that specifically recognizes aggregated α-synuclein. **(B)** Percentage of cells positive for α-syn aggregates were counted and values were compared to control (uninfected) cells. Data represent mean ± SEM. **(C)** Cells were infected with DSV and harvested for western blotting to probe for α-syn expression. Actin was used as loading control. **(D)** Western blot images were quantified using Fiji Image J. Data represent mean ± SEM as a fold change difference compared to control. Control values were set as 1. The blot shown is a representative image of multiple independent experiments that were conducted atleast three times. The blot was cut to allow probing for two proteins on the same gel, actin for the upper bands while the lower bands were used for probing synuclein.**p < 0.01, *p < 0.05.

α-synuclein is an intrinsically unstable protein and has a propensity to misfold and accumulate as protein aggregates. α-syn monomers can aggregate to form intermediate oligomers which further assemble into α-syn fibrils that form Lewy Bodies in PD patients (Henderson et al., 2019). Thus, monitoring increase in α-syn aggregates is a commonly used readout for studying the pathogenesis of PD. We first tested whether DSV could induce an increase in α-syn aggregation in intestinal enteroendocrine cells STC-1. These cells are known to produce and secrete α-syn constitutively (Chandra et al., 2017) and thus serve as good cellular models to test the possible effect of DSV on α-syn. We infected STC-1 with DSV at various multiplicity of infection (MOI) 40, 60 and 80 for 24 h (Figures 1A,B). Cells were processed for immunofluorescence using a conformation-specific antibody (Abcam) validated to bind to aggregated form of α-syn and which appears to have a lesser affinity for α -syn monomers. Using this antibody, we found a significant increase in the percentage of cells with α-syn aggregates in the presence of DSV when compared to uninfected cells (con) (DSV 60: 48.33% ± 7.26 vs. con: 7.12% ± 1.39, *p* < 0.01; DSV80: 45.7% ± 13.05 vs. con, *p* < 0.05; Figure 1B). In addition, the size of these aggregates also appeared bigger when compared to control cells. Next, we tested whether DSV caused an increase in α-syn protein expression. DSV (MOI 60) induced an increase in α-syn protein expression when compared to con (DSV: 2.07 ± 0.32 vs. con: 1.0, *p* < 0.01; Figures 1C,D). As STC-1 cells constitutively produce and secrete α-syn, we also observed basal levels of α-syn protein expression and few aggregates in control uninfected cells. Thus, our findings indicated that DSV increased both the expression and aggregation of α-syn in STC-1 cells.

### 3.2 DSV induced increase in extracellular level of α-syn protein into STC-1 culture supernatant

**Figure 2:**
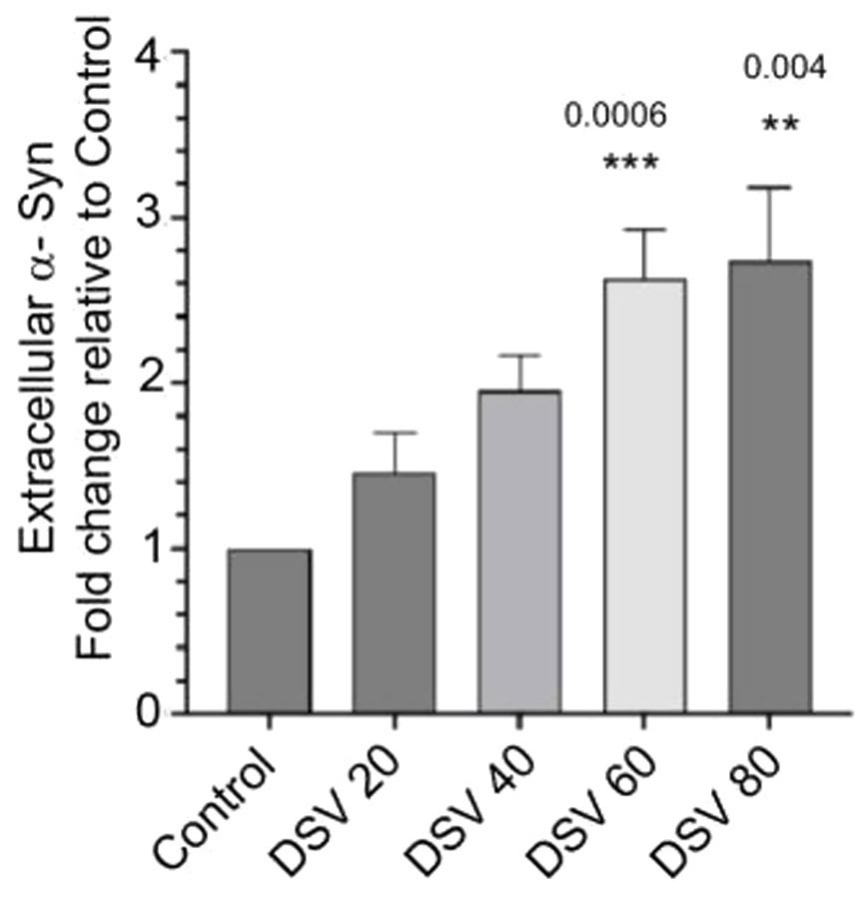
DSV induces increase in extracellular levels of synuclein in culture supernatants of STC-1 Cells were infected for 24 h. with various DSV MOIs. Culture supernatants were harvested and filtered to remove bacteria and the amount of extracellular synuclein was measured using ELISA kit using manufacturer’s protocol. Data represent mean ± SEM. Values were normalized against control uninfected cell supernatants. **p < 0.01, ***p < 0.001.

Next, we tested whether DSV increased the levels of α-syn in extracellular medium in STC-1 cells (Figure 2). Various α-syn-aggregation-inducing stimuli are known to cause increased α-syn secretion (Khalaf et al., 2014; Jang et al., 2010) which allows its propagation and cell–cell spread and thus enabling it to seed and form new aggregates of α-syn protein produced by recipient cells. We infected STC-1 cells with DSV for 24 h and collected culture supernatants (sup) at the end of the incubation. Sups were passed through 0.2 m filter to remove extracellular bacteria and any floating cells. The amount of extracellular α-syn was measured using ELISA. We observed a dose-dependent fold-change increase in α-syn concentration in sups of STC-1 cells infected with DSV when compared to sups from control cells. Significant increase in levels of α-syn were observed at higher MOI (DSV60: 2.62 ± 0.29 vs. con:1.0, *p* < 0.01; DSV80: 2.73 ± 0.44 vs. con, *p* < 0.01).

We also tested whether these effects were specific to DSV and compared our findings with DSV with another gram negative gut bacteria *B. thetaiotaomicron* (B. theta) that represent a major phyla Bacteroidota, predominant resident bacteria in the intestine. We treated STC-1 cells with either DSV or B. theta for 24 h. Cells were then processed for immunofluorescence using anti-α-syn aggregate specific antibody and cell culture sups were collected and passaged through 0.2 m filters. α-syn was measured in the culture sups by ELISA (Figure S1). We found no significant increase in percentage of cells with syn aggregates in response to B. theta when compared to control (Figure S1A). In contrast, DSV caused a significant increase in aggregates as observed in Figure 1. Similarly, no significant increase in extracellular α-syn was observed in cell culture sups of cells infected with B. theta compared to control cell sup (Figure S1B). These findings suggest that the effects on α-syn in our study were specific to DSV.

### 3.3 DSV-infected STC-1 sup induced increase in α-syn aggregates in neuronal Sy5y cells

**Figure 3:**
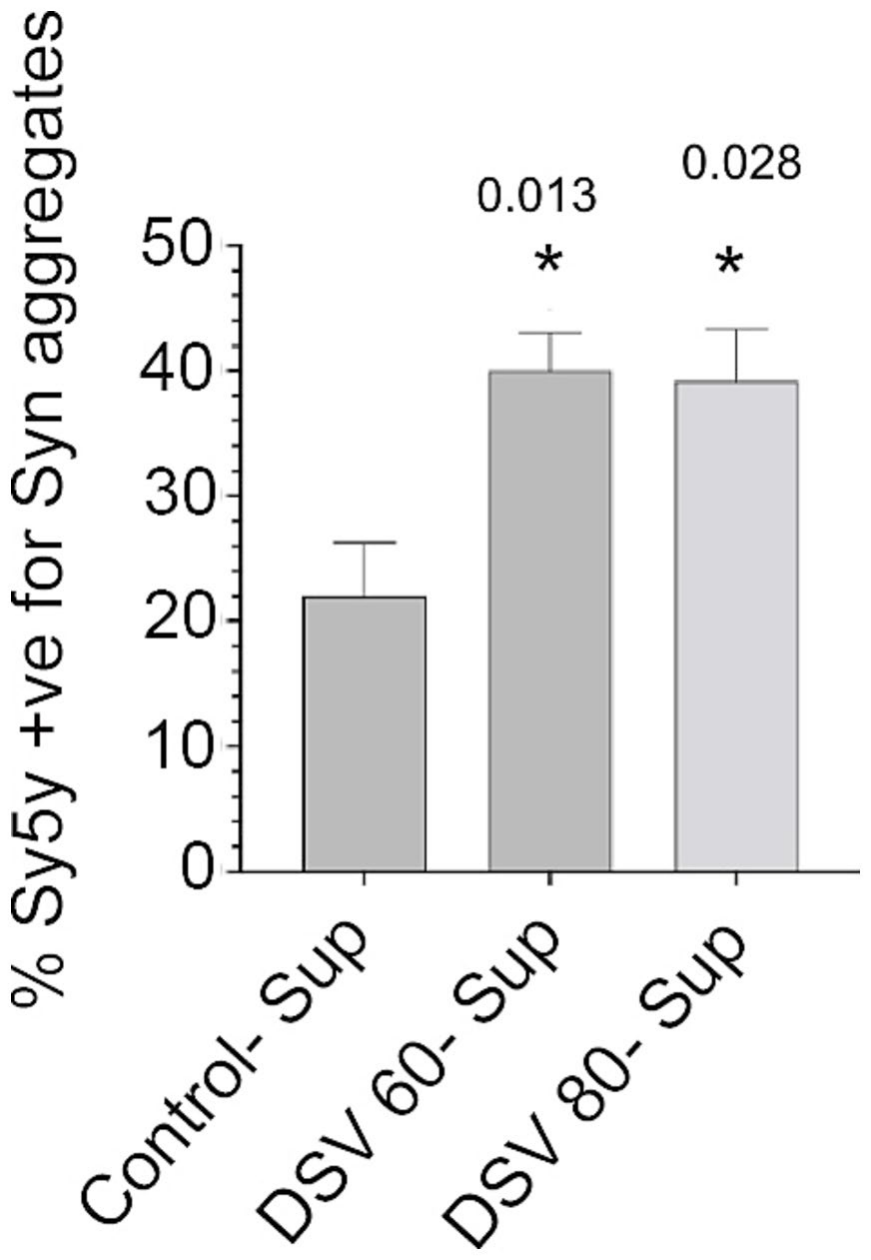
Sup from DSV-infected STC-1 induce increase in synuclein aggregate in neuronal Sy5y cells STC-1 cells were infected for 24 h. with various DSV MOIs. Culture supernatants (sup) were through 0.2 μm filters and added to Sy5y cells, for 24 h. Sy5y cells were then fixed and processed for immunofluorescence using α-synuclein aggregate antibody. Percentage of cells positive for aggregates were counted and values were compared to cells treated with control uninfected sup from STC-1 cells. Data represent mean ± SEM. A two-tailed students t-test was used to analyze the statistical difference between the groups. *p < 0.05.

In the subsequent experiment, we investigated whether sup of DSV-infected STC-1 cells could further induce α-syn aggregation in neuronal Sy5y cells (Figure 3). We found that filtered sup from DSV-infected STC-1, that contained significantly higher levels of α -syn compared to control cell sups (as shown in Figure 2), induced α -syn aggregation in Sy5y cells (DSV60-sup: 40.05 ± 2.95; DSV80-sup: 39.18 ± 4.18; con-sup: 22.00 ± 4.27, *p* < 0.05, compared to Con-sup). We also confirmed the increase in α-syn in sups from DSV-infected STC-1 cells in the same experiment. These results suggest that DSV is capable of causing α-syn aggregation in neuronal cells indirectly via extracellular release of one or more “aggregation-inducing signal(s)” by intestinal cells. As α-syn is known to seed more aggregates in recipient cells via cell-to-cell spread (Hoffmann et al., 2019), our results suggest that α-syn released by DSV-infected cells may be responsible for causing increased α -syn aggregation in neuronal cells, even in the absence of the bacteria. However, it is also possible that extracellular signals other than α-syn from DSV-infected STC-1 sup may be driving α-syn aggregation in Sy5y cells. Additionally, whether the α-syn aggregates observed in Sy5y cells were endogenous or whether some of these aggregates were taken up from DSV-infected sup is not clear.

### 3.4 DSV directly induced increased α-syn aggregation in Sy5y

**Figure 4:**
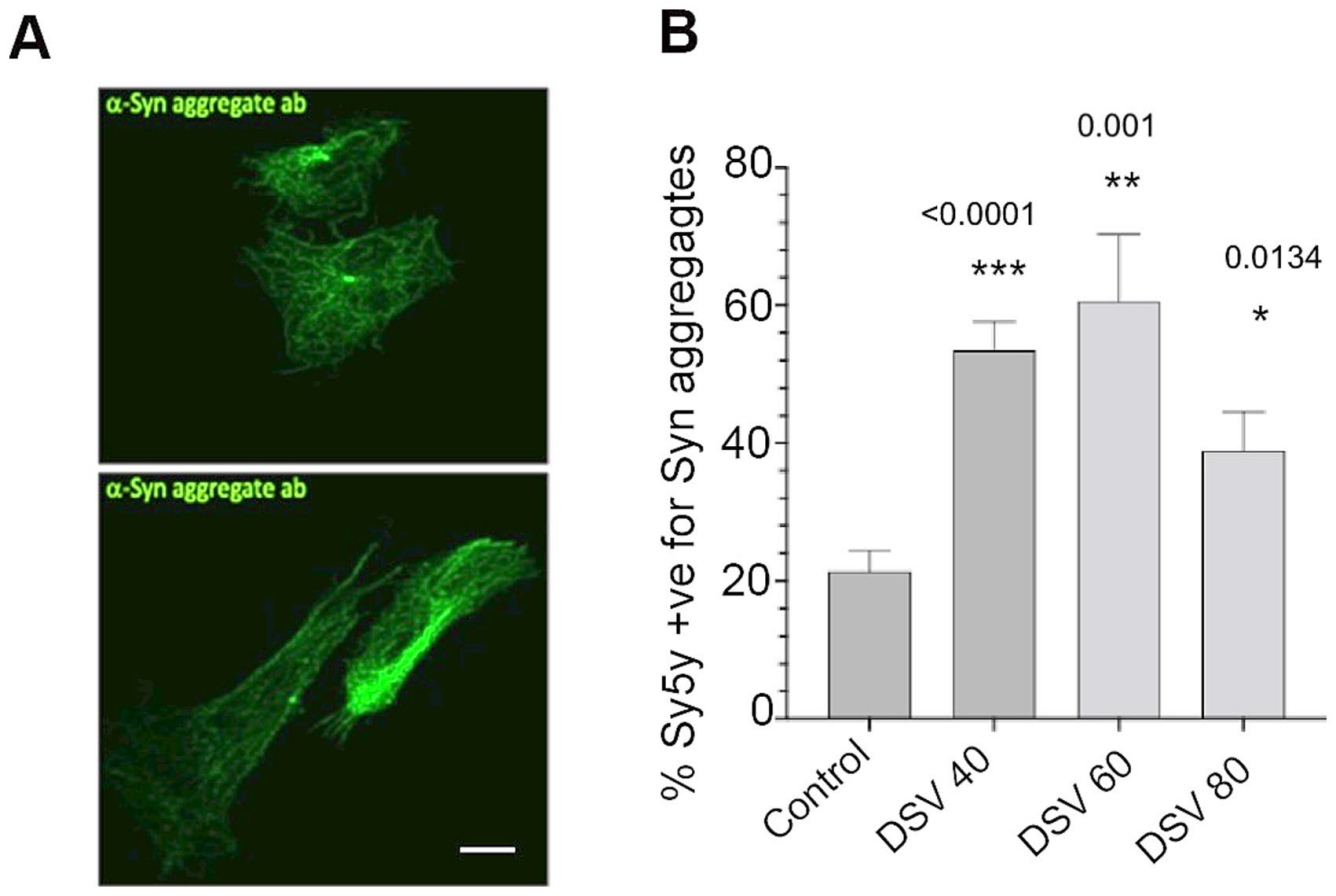
DSV directly induces increase in α-synuclein aggregates in Sy5y neuronal cells **(A)**. Cells were infected for 24 h. with various DSV MOIs. Cells were fixed and processed for immunofluorescence using antibody that recognizes aggregated α-synuclein. **(B)** Percentage of cells positive for aggregates were counted and values were compared to control uninfected cells. Data represent mean ± SEM. A two-tailed students *t*-test was used to analyze the statistical difference between control and DSV-infected cells. ***p < 0.001, **p < 0.01, *p < 0.05.

We also tested whether DSV could directly induce increase in the number of neuronal cells with α -syn aggregates (Figure 4). It is possible that in the setting of dysbiosis, DSV in the gut could access neuronal cells through leaky gut (induced by DSV, among other factors) either by way of vagus nerve or through circulation. As such, presence of a variety of bacteria including Proteobacteria has been reported in the brain of PD patients (Pisa et al., 2020) and translocation of gut bacteria to the brain has also been reported (Thapa et al., 2023). Thus, we infected neuronal Sy5y cells with DSV at various MOI to test whether infection of these cells directly with DSV could elicit a similar response as the STC-1. We found that DSV directly induced a significant increase in the number of Sy5y cells with α -syn aggregates even at a lower MOI 40 (DSV40: 53.46 ± 4.20 vs. con: 21.43 ± 2.94, *p* < 0.01; DSV60: 60.57 ± 9.71 vs. con, *p* < 0.01; DSV80: 38.92 ± 5.60, *p* < 0.05). (Figures 4A,B). These results indicate that even lower number of DSV are potent in inducing α-syn aggregation directly in neurons.

### 3.5 DSV inhibited tyrosine hydroxylase (TH) protein expression in Sy5y cells

**Figure 5:**
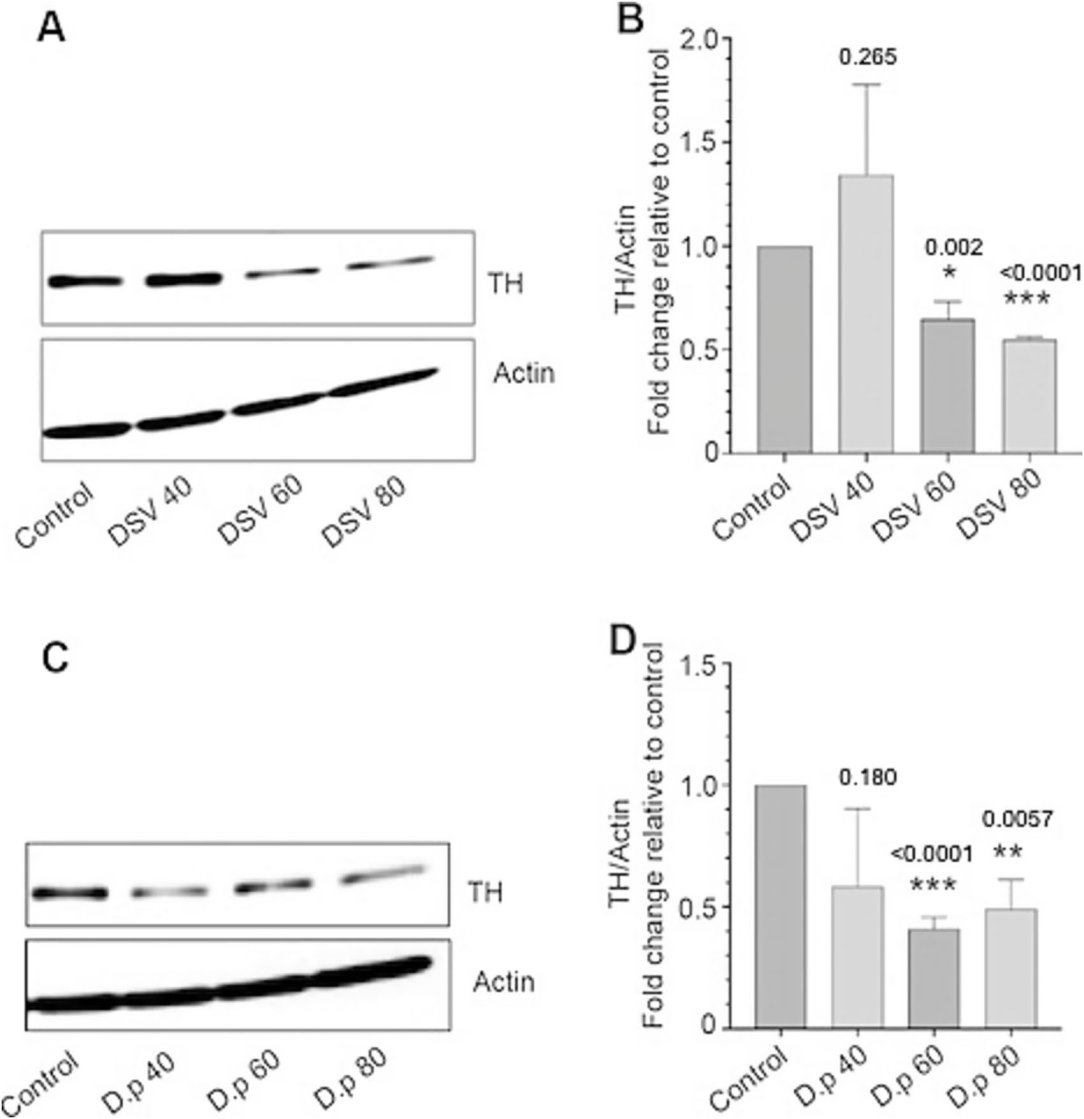
*Desulfovibrio* inhibits tyrosine hydroxylase (TH) protein expression in neuronal Sy5y cells **(A,B)**. Cells were infected for 24 with DSV at various MOIs. Cells were harvested for western blotting to probe for TH expression. Actin was used as a loading control. Data represent mean ± SEM as a fold change difference compared to control. **(C,D)** Cells were infected for 24 with *D. piger* at various MOIs. Cells were harvested for western blotting to probe for TH expression. Western blot images were quantified using Fiji Image J. Data represent mean ± SEM as a fold change difference compared to control. Control values were set as 1. A two-tailed student t-test was used to analyze the statistical difference between control and DSV-infected cells. ***p < 0.001, **p < 0.01, *p < 0.05.

Next, we tested whether DSV inhibited TH expression in Sy5y cells. Cells were infected with DSV and processed for Western blotting to probe for TH expression. Approximately, 35–40% inhibition was observed in TH expression in DSV-infected cells (DSV60: 0.65 ± 0.01vs con: 1.0, *p* < 0.05; DSV80: 0.59 ± 0.01, *p* < 0.001) when compared to control uninfected cells (Figures 5A,B). As α -syn has been reported to inhibit TH (Perez et al., 2002; Yu et al., 2004), it is possible that DSV may inhibit this rate-limiting enzyme via upregulation of α-syn. Alternatively, DSV may inhibit TH expression by a mechanism independent of α-syn. To further validate our findings, we infected Sy5y cells with *D. piger* (D. p), a species of *Desulfovibrio* that are found in patients with PD (Huynh et al., 2023). D.p is a clinically significant bacteria linked to diseases (Singh et al., 2023). We found that similar to DSV, D.p significantly inhibited TH expression in Sy5y cells when compared to control cells (D.p 60: 0.41 ± 0.04 vs. con:1.0, *p* < 0.001; D.p 80: 0.49 ± 0.12, *p* < 0.01). Thus, our results suggest that the *Desulfovibrio spps*. Are capable of inhibiting the expression of TH, a rate-limiting enzyme involved in dopamine synthesis.

### 3.6 Effect of DSV on α-synuclein in intestinal tissues, plasma, and in mid-brain

**Figure 6:**
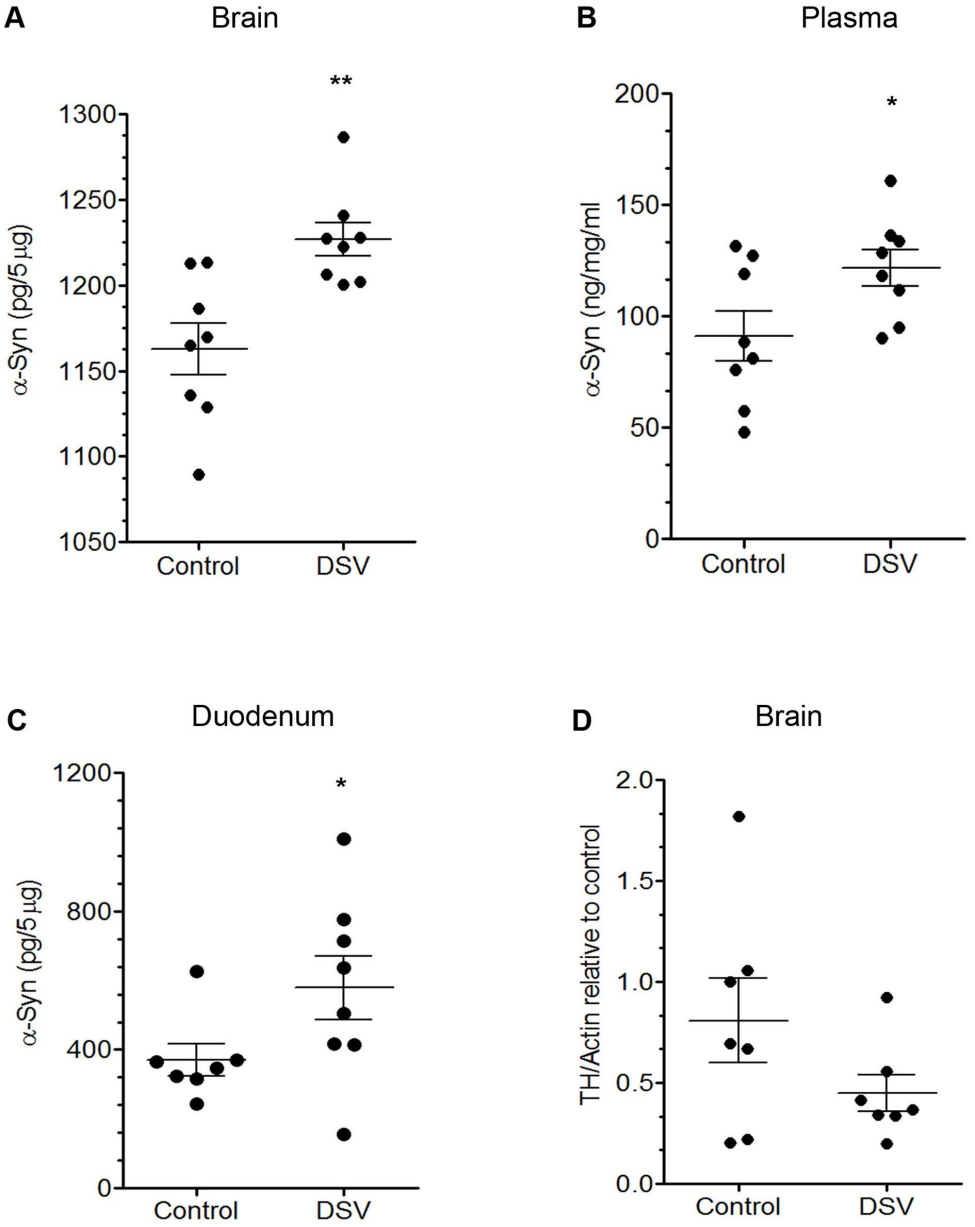
DSV increase α-syn levels in animal tissues Mice were gavaged with either DSV (10^9^) or lactulose vehicle control for 24 h. Mice were then euthanized and intestinal tissues, brain tissue, and plasma were collected. **(A)** Level of α-syn in mid-brain lysates (in 5 μg lysates), **(B)** in proximal small intestine corresponding to duodenum (in 5 μg lysates) and in, **(C)** plasma (1:2000 dilution), as measured by ELISA. Protein concentration in plasma sample was determined by Bradford reagent to normalize the results. **(D)** TH expression in brain lysates in control vs. DSV animals. Forty micrograms Lysates were run on SDS-PAGE and analyzed by Western blotting using TH antibody. Actin was used as a loading control. Images were quantified with Fiji Image J. All data represent mean ± SEM as a fold change difference compared to control. *p < 0.05.

Lastly, we investigated whether some of our findings in cell lines could be confirmed in animals (Figure 6). Mice were orally gavaged with DSV for 24 h following which brain and intestinal tissues corresponding to duodenum, ileum, and colon were collected and analyzed. Plasma samples were also collected. For brain, tissue samples were collected from the mid-brain to analyze synuclein as well as TH expression in this section as it is enriched in dopamine-producing neurons (in substantia nigra). We found that in mice gavaged with DSV, a small but significant increase in levels of α-syn in brain lysates was observed when compared to control animals (DSV: 1227 ± 9.91 pg./5 mg vs. Control: 1163 ± 15.19 pg./5 mg, *p* < 0.01; Figure 6A). It is noteworthy that while 2 out of 8 control animals showed values above 1,200 pg., all 8 animals in DSV gavage group exhibited values higher than 1,200 pg. suggesting that DSV gavage for 24 h initiated a cascade causing an increase in α-syn in the mid-brain section. Next, we measured α-syn in plasma samples of control and DSV-gavage group. A significant increase was observed in α-syn in plasma samples of DSV-gavaged mice when compared to control mice (DSV: 122.0 ± 8.19 ng/mL vs. Con: 91.25 ± 11.25 ng/mL, *p* < 0.05; Figure 6B) implicating that DSV induced release of α-syn into the plasma from the intestine. In order to confirm that production of α-synuclein induced by DSV in plasma and brain originates in the gut, we measured α-syn levels in the proximal region of the intestine corresponding to duodenum, which is also a site where high concentration of α-syn expressing enteroendocrine cells (EEC) are found (Chandra et al., 2017). DSV-gavaged mice had significantly higher level of α-syn (in pg./5 mg) in duodenum when compared to control mice (DSV: 638.6 ± 81.54 vs. Control: 369.7 ± 45.64, *p* < 0.05; Figure 6C). As some EEC are also found in ileum and colon, we checked the level of α-syn in these tissues. However, we did not find increase in α-syn levels in the ileum or the colon in DSV group when compared to control (data not shown) suggesting that 24 h DSV gavage significantly induced α-syn only in the duodenum tissue.

Next, we compared TH expression in the brain lysates of control and DSV-gavaged animals by Western blotting. We found ~2 fold decrease in TH expression in DSV-gavaged group when compared to control mice (DSV: 0.448 ± 0.08 vs. Con: 0.808 ± 0.21, *p* > 0.05; Figure 6D). While this inhibition was not statistically significant, there was a trend pointing to a lower level of TH expression with DSV vs. control.

Thus far, our animal data suggest that DSV could initiate a response in intestinal and brain tissues similar to what we observed *in vitro*.

## 4 Discussion

In this study, we demonstrated that *Desulfovibrio vulgaris* induced increased α-syn aggregation directly in intestinal and neuronal cells. In addition, the conditioned medium from DSV-infected intestinal cells induced α-syn aggregates in neuronal cells. DSV also inhibited TH expression in neuronal cells. Moreover, we found that DSV administered into the gut increased α-syn levels in the proximal but not distal gut and also increased the concentration of α-syn in plasma and in the brain.

Accumulating evidence suggest that PD originates in the gut and that gut-brain axis plays an important role in disease development (Braak et al., 2003; Liddle, 2018). We have previously reported two primary hypotheses that suggest either α-syn aggregation occurs in the gut and spreads to the brain via a neuronal pathway or systemic pathway, or gastrointestinal dysfunction contributes to the pathophysiology of the disease via systemic influences and prodromal gastrointestinal symptoms often occur years before the onset of active disease in PD patients (Ryman et al., 2023). **S**tudies have also observed accumulation of α-syn in intestinal biopsies in early stages of PD patients (Hilton et al., 2014; Shannon et al., 2012). Importance of gut-brain axis in the pathogenesis of PD was demonstrated by vagotomy in animals which led to decrease in α-syn accumulation in brain (Kim et al., 2019) and in human subjects where truncal vagotomy appeared to lower the risk of PD (Svensson et al., 2015). Many studies have reported gut microbial dysbiosis in PD patients (Zhang et al., 2020; Zhang et al., 2023; Huang et al., 2021). *Desulfovibrio* is one of the most abundant genus among sulfate reducing bacteria that produce hydrogen sulfide (H_2_S). These bacteria are normally present as minor members of gut microbial community in a healthy human gastrointestinal tract. However, in the setting of dysbiosis, loss of diversity occurs leading to loss of beneficial microbes and overgrowth of opportunistic pathobionts such as *Desulfovibrio*. In keeping with this, many diseases that are associated with microbial dysbiosis often present with overgrowth of *Desulfovibrio* (Singh et al., 2023). This is also observed in case of PD where an increase in the density of *Desulfovibrio* occurs in fecal samples is observed in PD patients (Murros et al., 2021; Nie et al., 2023; Li et al., 2023). A more direct link between *Desulfovibrio* and PD pathogenesis was reported by Huynh et al. (2023) who isolated these bacteria from stool samples of PD patients or healthy individuals and fed them to *C. elegans*. It was found that worms that were fed *Desulfovibrio* from PD patients or healthy individuals exhibited significantly higher numbers of α -syn aggregates in their head regions when compared to worms that were fed *E. coli* negative control suggesting that *Desulfovibrio* induces α-syn aggregate formation. However, it remained unclear whether these bacteria induced α-syn aggregates first in the gut which then traveled to the brain or the bacteria directly accessed the brain in *C. elegans* and induced α-syn aggregates locally. Using *in vitro* cellular model system, our study demonstrates that *Desulfovibrio* is capable of inducing α-syn aggregates both in the intestinal and in neuronal cells and can also indirectly transmit the α-syn- aggregation signal from intestinal cells to neuronal cells.

How DSV causes α-syn aggregation remains to be explored. We observed that DSV also induced protein expression of α-syn. Thus, increase in α-syn aggregates could be due to upregulation of α -syn protein expression by DSV. While it remains speculative at this point, we propose few mechanisms by which DSV could increase synuclein expression and aggregation. One such mechanism is that increase in protein expression and aggregation of α-syn by DSV could be mediated by activation of toll-like receptor −2 (TLR-2) by DSV. The basis for this possibility is that our previous study showed that DSV activated proinflammatory pathway in immune cells in a TLR-2 dependent manner. TLR-2 expression is upregulated in PD postmortem brains and blood (Drouin-Ouellet et al., 2014; Dzamko et al., 2017). Activation of TLR-2 has been shown to increase the expression of α-syn protein and its accumulation in aggregates (Dzamko et al., 2017; Kim C. et al., 2015). TLR-2/Myd88/NFkB pathway was also found to be involved in α-syn spreading (Dutta et al., 2021). Moreover, TLR-2 antagonists inhibited release of α-syn from STC-1 cells (Hurley et al., 2023). In addition, Anti-TLR2 therapy has been reported to reduce α-syn aggregation and to ameliorate neurodegeneration (Kim et al., 2018). STC-1 express TLR-2 among other TLRs (Bogunovic et al., 2007). Based on these evidence it is possible that DSV may mediate its effects on α-syn in TLR-2 dependent manner.

Another potential mechanism by which DSV may lead to accumulation of α-syn aggregates is by inhibiting their degradation by cellular mechanisms such a lysosomal degradation (Lee et al., 2004), leading to dysfunctional clearance of α-syn aggregates. These mechanisms remain speculative and require direct experimental validation. Future studies will be aimed at investigating these potential cellular mechanisms that may be employed by DSV to induce α-syn expression and aggregation.

On the bacterial end, it is plausible that DSV may induce increase in α-syn aggregates via a potential bacterial amyloid protein in a manner similar to that of *E. coli* Curli protein (Sampson et al., 2020). In support of this argument, it was recently predicted that DSV may produce amyloid fibril proteins (Kallberg et al., 2001). DSV is known to produce H_2_S which may possibly contribute in the α-syn aggregate formation by releasing cytochrome C (Cyt C) from mitochondria which has been shown to cause oligomerization of α -syn (Murros, 2022). As a Gram- negative bacteria, DSV produces lipopolysaccharide (LPS). LPS from *E. coli* could induce α -syn aggregation (Kim et al., 2016) and cause neuroinflammation which is an underlying mechanism of PD (Liu and Bing, 2011). Thus, it is possible that LPS produced by DSV may elicit similar responses. Thus, DSV may potentially trigger α-syn aggregation by these bacterial mediators.

Next, we reported that DSV caused release of α-syn from STC-1 cells and that applying conditioned medium from DSV-infected cells induced α-syn aggregation in Sy5y neuronal cells. The molecular mechanism underlying these effects remain to be defined. α-syn has been reported to be secreted by mechanisms such as exosome vesicle secretion and exocytosis (Lee et al., 2005; Emmanouilidou et al., 2010; Alvarez-Erviti et al., 2011). In addition, α -syn may be released by cells as a result of injury or cell death (Tsigelny et al., 2008). H_2_S-induced release of Cyt C could lead to apoptosis and release of α -syn aggregates from cells (Ito et al., 2023). Whether DSV induces exocytosis, Cyt C release, or apoptosis of STC-1 cells remains to be determined. Another potential mechanism could be that α-syn secretion caused by DSV may spread to Sy5y cells through intercellular transfer or cell-to-cell spread through tunnel nanotubes (Dieriks et al., 2017) or may be endocytosed by recipient cells and cause further seeding of endogenous α-syn (Lee et al., 2010). Moreover, α-syn aggregation in Sy5y may occur through other yet unknown factors such as proinflammatory cytokines including TNF-a that may be present in the STC-1 Sup (Bogunovic et al., 2007). TNF-a is involved in syn aggregation and propagation as well as in neuroinflammation and plays an important role in the pathogenesis of PD (Bae et al., 2022; Cheng et al., 2022). Based on our previous observation that DSV activates TNF-a (Singh et al., 2024), We postulate that that DSV may cause activation and secretion of TNF-a in STC-1 as an underlying mechanism of α-syn aggregation in Sy5y mediated by DSV- infected STC-1 sup. Whether or not, DSV could mediate all these proposed mechanisms remain to be explored.

In the context of PD, the question of how extracellular α-syn from DSV-infected intestinal cells is transported to the brain is central to understanding the spread of this disease from the intestine to the brain. A study showed that vagotomy prevented spread of α-syn fibrils from intestine to the brain underscoring the important role of vagus nerve in transporting α-syn through gut-brains axis. Additionally, increased α-syn levels have been detected in the plasma of PD patients (El-Agnaf et al., 2003; Lee et al., 2006) suggesting that syn aggregates may also be transported through bloodstream. Our previous study found that DSV causes increase in intestinal epithelial cell permeability in snail-dependent manner (Singh et al., 2022). Thus, is it possible that DSV-induced leaky gut may allow translocation of bacteria or their products as well as α-syn from gastrointestinal tract into the circulation which carries these mediators to the brain. Our animal data showing increased α-syn levels in plasma in DSV-gavaged animals support this possibility. As snail is also responsible for causing disruption of blood brain barrier (BBB; Kim B. J. et al., 2015), it is feasible that DSV may cause BBB permeability by activating snail thus directly accessing neuronal cells. Translocation of gut bacteria to the brain through vagus nerve in the setting of dysbiosis has also been suggested (Thapa et al., 2023). Moreover, presence of bacteria in the brain of PD patients was reported (Pisa et al., 2020). Thus, it is possible that in the setting of dysbiosis such as in PD where DSV bloom may occur, DSV may translocate to the brain. Together, this highlights two potential pathways, either via the vagus nerve or via systemic circulation that will be evaluated in future studies.

We found that DSV also downregulated the protein expression of TH, a rate- limiting enzyme involved in the first step of dopamine synthesis where it hydroxylates L-tyrosine to L-dihydroxyphenylalanine (L-DOPA). Loss of dopaminergic neurons and decreased dopamine production is a hallmark feature of PD. It has been shown that PD patients have a decreased expression of TH (Rausch et al., 2022; Kawahata and Fukunaga, 2020; Nakashima et al., 2009). We found that *D. vulgaris* as well as PD associated *D. piger* caused inhibition of protein expression of TH in Sy5y cells. As α-syn binds to TH and suppress its activity (Perez et al., 2002). While it remains to be tested, we propose that DSV could inhibit TH expression via upregulation of and enhanced binding of α-syn to TH. It also remains unclear whether DSV affects the TH activity and its phosphorylation which is important for its activity (Dunkley et al., 2004). Moreover, it is possible dopamine levels and localization within the neurons may be impaired by DSV via its effects on TH. These queries warrant further investigation into the role of DSV as a causal gut bacteria that contributes to the development of PD.

Finally, we confirmed some of our *in vitro* findings by orally gavaging mice with DSV for 24 h. We found that DSV-gavaged mice showed a small but significant increase in the level of α-syn in duodenal tissue lysates, in plasma, and in mid-brain lysates. A trend toward decrease in TH expression in brain samples was also observed in DSV-gavaged animals compared to control mice. The rationale for using 24 h time point for gavage came from our previous studies that demonstrated that a short period of oral gavage of DSV (under 24 h) is sufficient to significantly alter various pathways in the intestine and also brain physiology including impairing working memory in mice and inducing anxiety-like behavior (Ritz et al., 2016; Ritz et al., 2017; Singh et al., 2021; Coffman et al., 2024). These studies suggested that once administered, DSV acts rapidly in causing physiological and behavioral changes in mice. In this study, we showed that DSV gavage for 24 h is impactful and elicits potentially early stages of changes pertaining to α-syn in the intestine and in the brain. It also mimics the time span we used to infect STC-1 with DSV *in vitro*. Interestingly, we observed that in this duration, DSV only increased α-syn levels in duodenal section of small intestine and not in ileum or in colon. In health, the microbiota of the gastrointestinal tract is largely contained in the distal gut and that the concentration of bacteria drops from 10^12^/g in the colon to 10^3^/g in the duodenum (Levitt, 1969). Since DSV is a resident gut bacteria, limiting the α-syn response to the proximal but not distal gut would restrict the response to DSV, i.e., increased production of α-syn, to the pathologic state of small intestinal bacterial overgrowth where the gut microbiota expands proximally from the distal gut into the proximal gut.

As there was a small but significant increase in α-syn in plasma and brain, α-syn induction by DSV in duodenum was sufficient to elicit downstream events leading to an increase in α-syn in the brain of DSV-gavaged mice. The possibility of transport of α-syn from duodenum to the brain through circulation was also suggested by the finding of increased concentration of α-syn in plasma. We also observed a trend toward decrease in brain TH levels in DSV-gavaged animals. However, this difference was statistically insignificant. We recognize that a 24 h post-gavage interval may not be sufficient to observe a significant inhibitory effects of DSV on TH and that a longer administration time may be required to elicit a more robust inhibition of TH (and induction of α-syn) by DSV. Future studies will be aimed at increasing the time of exposure from 24 h to DSV to longer durations to analyze the kinetics of synuclein aggregation, its spread and TH expression to better understand the role of DSV in synucleopathy.

A limitation of our study is that we did not explore the mechanisms by which DSV increases α-syn, mediates release of α-syn, causes aggregation of synuclein in Sy5y cells in response to STC-1 conditioned medium, and inhibits TH expression. Further investigation is needed to explore the role of DSV gavage on α-syn in the intestine and brain by immunohistochemistry. In addition, we did not include either dead DSV or another negative control bacteria in gavage experiments to investigate whether the observed effects were exclusive to live DSV. Future *in vitro* and *in vivo* studies will be aimed at addressing these questions and testing the discussed speculated mechanisms in order to understand comprehensively howDSV contributes to pathophysiology of PD by affecting α-syn and TH function. Moreover, longer post-gavage times (and possibly multiple gavages) would be important to understand the chronic effects of DSV in the disease development.

**Figure 7:**
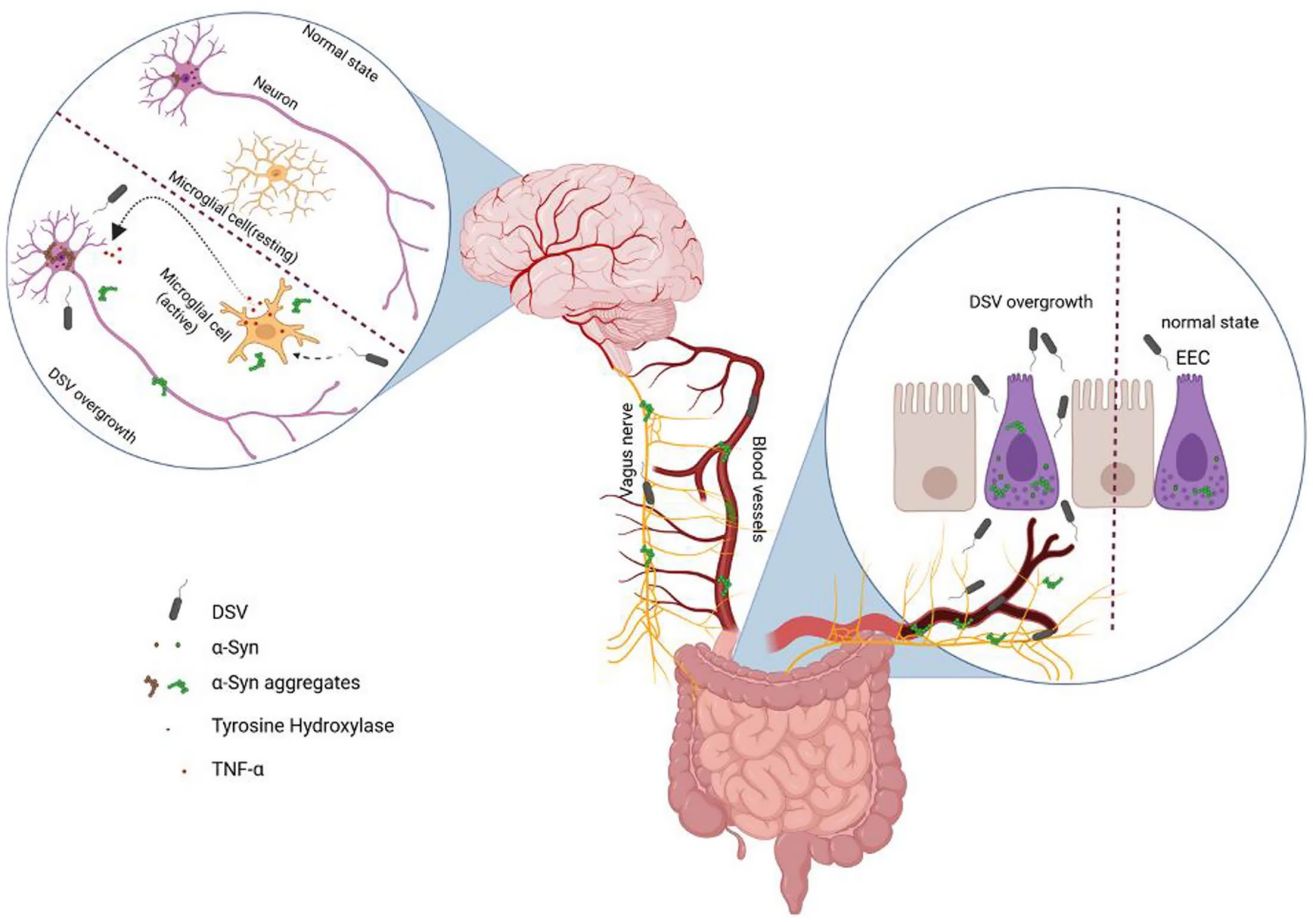
Schematic representation of how DSV may be contributing to the pathogenesis of PD. In the setting of dysbiosis, DSV overgrowth may occur in the intestine. Higher number of DSV could then induce increase in α-synuclein aggregates in the intestinal cells such as enteroendocrine cells (EEC) that are known to produce α-syn. In addition, DSV causes secretion of α-syn aggregates from EEC. DSV is also known to disrupt intestinal tight junction barrier leading to leaky gut which, in turn, leads to the translocation of bacteria in the bloodstream. Extracellular α-synuclein aggregates along with DSV could be carried by vagus nerve and/or blood circulation to the brain. In the brain, the transported α-synuclein may further spread to neurons and seed endogenous α-syn aggregation in these cells. Gut-derived α-syn could also activate microglia to produce and secrete TNF-a which in turn could induce α-syn aggregation in the neurons. Furthermore, DSV translocated through leaky gut could directly access neurons to cause α-syn aggregation in these cells. DSV may also activate microglia to secrete TNF-a which in turn could induce α-syn aggregation in neuronal cells. In the neurons, DSV further directly inhibits tyrosine hydroxylase (TH) either by an unknown mechanism and/or mediates its inhibitory effects on TH via its induction of α-syn. Figure was generated using Biorender.com.

In conclusion, our study shows for the first time that *Desulfovibrio* bacteria could induce increase in α -syn aggregates in intestinal STC-1 cells and in neuronal Sy5y cells either directly or indirectly through intestinal cells and also inhibit tyrosine hydroxylase protein expression in neuronal cells. Our preliminary animal data also supports our *in vitro* outcomes. Based on our overall findings, we propose a model of how DSV may be contributing to the development and pathogenesis of PD (Figure 7). Taken together, our findings provide a mechanistic causal link between SRB and PD and identify novel therapeutic targets for mitigating SRB effects in the pathogenesis of PD.

## Data Availability

The original contributions presented in the study are included in the article/Supplementary material, further inquiries can be directed to the corresponding author.

## References

[ref1] Alvarez-Erviti L. Seow Y. Schapira A. H. Gardiner C. Sargent I. L. Wood M. J. . (2011). Lysosomal dysfunction increases exosome-mediated alpha-synuclein release and transmission. Neurobiol. Dis. 42, 360–367. doi: 10.1016/j.nbd.2011.01.029, 21303699 PMC3107939

[ref2] Baba M. Nakajo S. Tu P. H. Tomita T. Nakaya K. Lee V. M. . (1998). Aggregation of alpha-synuclein in Lewy bodies of sporadic Parkinson's disease and dementia with Lewy bodies. Am. J. Pathol. 152, 879–884. 9546347 PMC1858234

[ref3] Bae E. J. Choi M. Kim J. T. Kim D. K. Jung M. K. Kim C. . (2022). TNF-alpha promotes alpha-synuclein propagation through stimulation of senescence-associated lysosomal exocytosis. Exp. Mol. Med. 54, 788–800. doi: 10.1038/s12276-022-00789-x, 35790884 PMC9352737

[ref4] Bartl M. Xylaki M. Bahr M. Weber S. Trenkwalder C. Mollenhauer B. (2022). Evidence for immune system alterations in peripheral biological fluids in Parkinson's disease. Neurobiol. Dis. 170:105744. doi: 10.1016/j.nbd.2022.105744, 35513230

[ref5] Beach T. G. Adler C. H. Sue L. I. Vedders L. Lue L. White C. L. III . (2010). Multi-organ distribution of phosphorylated alpha-synuclein histopathology in subjects with Lewy body disorders. Acta Neuropathol. 119, 689–702. doi: 10.1007/s00401-010-0664-3 20306269 PMC2866090

[ref6] Bogunovic M. Dave S. H. Tilstra J. S. Chang D. T. Harpaz N. Xiong H. . (2007). Enteroendocrine cells express functional toll-like receptors. Am. J. Physiol. Gastrointest. Liver Physiol. 292, G1770–G1783. doi: 10.1152/ajpgi.00249.2006 17395901 PMC3203538

[ref7] Braak H. Del Tredici K. Rub U. De Vos R. A. Jansen Steur E. N. Braak E. (2003). Staging of brain pathology related to sporadic Parkinson's disease. Neurobiol. Aging 24, 197–211. doi: 10.1016/s0197-4580(02)00065-9, 12498954

[ref8] Chandra R. Hiniker A. Kuo Y. M. Nussbaum R. L. Liddle R. A. (2017). Alpha-synuclein in gut endocrine cells and its implications for Parkinson's disease. JCI Insight 15:e92295. doi: 10.1172/jci.insight.92295 PMC5470880 28614796

[ref9] Cheng F. Fransson L. A. Mani K. (2022). Complex modulation of cytokine-induced alpha-synuclein aggregation by glypican-1-derived heparan sulfate in neural cells. Glycobiology 32, 333–342. doi: 10.1093/glycob/cwab126, 34939110 PMC8970428

[ref10] Coffman C. N. Carroll-Portillo A. Alcock J. Singh S. B. Rumsey K. Braun C. A. . (2024). Magnesium oxide reduces anxiety-like behavior in mice by inhibiting sulfate-reducing Bacteria. Microorganisms 12:1429. doi: 10.3390/microorganisms12071429, 39065198 PMC11279233

[ref11] Dieriks B. V. Park T. I. Fourie C. Faull R. L. Dragunow M. Curtis M. A. (2017). Alpha-synuclein transfer through tunneling nanotubes occurs in SH-SY5Y cells and primary brain pericytes from Parkinson's disease patients. Sci. Rep. 7:42984. doi: 10.1038/srep42984, 28230073 PMC5322400

[ref12] Drouin-Ouellet J. ST-amour I. Saint-Pierre M. Lamontagne-Proulx J. Kriz J. Barker R. A. . (2014). Toll-like receptor expression in the blood and brain of patients and a mouse model of Parkinson's disease. Int. J. Neuropsychopharmacol. 18:pyu103. doi: 10.1093/ijnp/pyu103, 25522431 PMC4438545

[ref13] Dunkley P. R. Bobrovskaya L. Graham M. E. Von nagy-Felsobuki E. I. Dickson P. W. (2004). Tyrosine hydroxylase phosphorylation: regulation and consequences. J. Neurochem. 91, 1025–1043. doi: 10.1111/j.1471-4159.2004.02797.x, 15569247

[ref14] Dutta D. Jana M. Majumder M. Mondal S. Roy A. Pahan K. (2021). Selective targeting of the TLR2/MyD88/NF-kappaB pathway reduces alpha-synuclein spreading *in vitro* and *in vivo*. Nat. Commun. 12:5382. doi: 10.1038/s41467-021-25767-1 34508096 PMC8433339

[ref15] Dzamko N. Gysbers A. Perera G. Bahar A. Shankar A. Gao J. . (2017). Toll-like receptor 2 is increased in neurons in Parkinson's disease brain and may contribute to alpha-synuclein pathology. Acta Neuropathol. 133, 303–319. doi: 10.1007/s00401-016-1648-8, 27888296 PMC5250664

[ref16] El-Agnaf O. M. Salem S. A. Paleologou K. E. Cooper L. J. Fullwood N. J. Gibson M. J. . (2003). Alpha-synuclein implicated in Parkinson's disease is present in extracellular biological fluids, including human plasma. FASEB J. 17, 1945–1947. 14519670 10.1096/fj.03-0098fje

[ref17] Emmanouilidou E. Melachroinou K. Roumeliotis T. Garbis S. D. Ntzouni M. Margaritis L. H. . (2010). Cell-produced alpha-synuclein is secreted in a calcium-dependent manner by exosomes and impacts neuronal survival. J. Neurosci. 30, 6838–6851. doi: 10.1523/JNEUROSCI.5699-09.2010, 20484626 PMC3842464

[ref18] Fasano A. Visanji N. P. Liu L. W. Lang A. E. Pfeiffer R. F. (2015). Gastrointestinal dysfunction in Parkinson's disease. Lancet Neurol. 14, 625–639. doi: 10.1016/S1474-4422(15)00007-1, 25987282

[ref19] Henderson M. X. Trojanowski J. Q. Lee V. M. (2019). Alpha-synuclein pathology in Parkinson's disease and related alpha-synucleinopathies. Neurosci. Lett. 709:134316. doi: 10.1016/j.neulet.2019.134316 31170426 PMC7014913

[ref20] Hilton D. Stephens M. Kirk L. Edwards P. Potter R. Zajicek J. . (2014). Accumulation of alpha-synuclein in the bowel of patients in the pre-clinical phase of Parkinson's disease. Acta Neuropathol. 127, 235–241. doi: 10.1007/s00401-013-1214-6, 24240814

[ref21] Hoffmann A. C. Minakaki G. Menges S. Salvi R. Savitskiy S. Kazman A. . (2019). Extracellular aggregated alpha synuclein primarily triggers lysosomal dysfunction in neural cells prevented by trehalose. Sci. Rep. 9:544. doi: 10.1038/s41598-018-35811-8, 30679445 PMC6345801

[ref22] Huang B. Chau S. W. H. Liu Y. Chan J. W. Y. Wang J. Ma S. L. . (2023). Gut microbiome dysbiosis across early Parkinson's disease, REM sleep behavior disorder and their first-degree relatives. Nat. Commun. 14:2501. doi: 10.1038/s41467-023-38248-4, 37130861 PMC10154387

[ref23] Huang Y. Liao J. Liu X. Zhong Y. Cai X. Long L. (2021). Review: the role of intestinal Dysbiosis in Parkinson's disease. Front. Cell. Infect. Microbiol. 11:615075. doi: 10.3389/fcimb.2021.615075, 33968794 PMC8100321

[ref24] Hurley M. J. Menozzi E. Koletsi S. Bates R. Gegg M. E. Chau K. Y. . (2023). Alpha-Synuclein expression in response to bacterial ligands and metabolites in gut enteroendocrine cells: an in vitro proof of concept study. Brain Commun. 5:fcad285. doi: 10.1093/braincomms/fcad285 37953845 PMC10636561

[ref25] Huynh V. A. Takala T. M. Murros K. E. Diwedi B. Saris P. E. J. (2023). Desulfovibrio bacteria enhance alpha-synuclein aggregation in a *Caenorhabditis elegans* model of Parkinson's disease. Front. Cell. Infect. Microbiol. 13:1181315. doi: 10.3389/fcimb.2023.1181315, 37197200 PMC10183572

[ref26] Ito N. Tsuji M. Adachi N. Nakamura S. Sarkar A. K. Ikenaka K. . (2023). Extracellular high molecular weight alpha-synuclein oligomers induce cell death by disrupting the plasma membrane. NPJ Parkinson's Dis. 9:139. doi: 10.1038/s41531-023-00583-0 37770475 PMC10539356

[ref27] Jang A. Lee H. J. Suk J. E. Jung J. W. Kim K. P. Lee S. J. (2010). Non-classical exocytosis of alpha-synuclein is sensitive to folding states and promoted under stress conditions. J. Neurochem. 113, 1263–1274. doi: 10.1111/j.1471-4159.2010.06695.x 20345754

[ref28] Kallberg Y. Gustafsson M. Persson B. Thyberg J. Johansson J. (2001). Prediction of amyloid fibril-forming proteins. J. Biol. Chem. 276, 12945–12950. doi: 10.1074/jbc.M010402200 11134035

[ref29] Kawahata I. Fukunaga K. (2020). Degradation of tyrosine hydroxylase by the ubiquitin-proteasome system in the pathogenesis of Parkinson's disease and Dopa-responsive dystonia. Int. J. Mol. Sci. 21:3779. doi: 10.3390/ijms21113779, 32471089 PMC7312529

[ref30] Khalaf O. Fauvet B. Oueslati A. Dikiy I. Mahul-Mellier A. L. Ruggeri F. S. . (2014). The H50Q mutation enhances alpha-synuclein aggregation, secretion, and toxicity. J. Biol. Chem. 289, 21856–21876. doi: 10.1074/jbc.M114.553297 24936070 PMC4139205

[ref31] Kim B. J. Hancock B. M. Bermudez A. Del cid N. Reyes E. Van Sorge N. M. . (2015). Bacterial induction of Snail1 contributes to blood-brain barrier disruption. J. Clin. Invest. 125, 2473–2483. doi: 10.1172/JCI74159, 25961453 PMC4497739

[ref32] Kim S. Kwon S. H. Kam T. I. Panicker N. Karuppagounder S. S. Lee S. . (2019). Transneuronal propagation of pathologic alpha-synuclein from the gut to the brain models Parkinson's disease. Neuron 103, 627–641 e7. doi: 10.1016/j.neuron.2019.05.035 31255487 PMC6706297

[ref33] Kim C. Lv G. Lee J. S. Jung B. C. Masuda-Suzukake M. Hong C. S. . (2016). Exposure to bacterial endotoxin generates a distinct strain of alpha-synuclein fibril. Sci. Rep. 6:30891. doi: 10.1038/srep30891 27488222 PMC4973277

[ref34] Kim C. Rockenstein E. Spencer B. Kim H. K. Adame A. Trejo M. . (2015). Antagonizing neuronal toll-like receptor 2 prevents synucleinopathy by activating autophagy. Cell Rep. 13, 771–782. doi: 10.1016/j.celrep.2015.09.044, 26489461 PMC4752835

[ref35] Kim C. Spencer B. Rockenstein E. Yamakado H. Mante M. Adame A. . (2018). Immunotherapy targeting toll-like receptor 2 alleviates neurodegeneration in models of synucleinopathy by modulating alpha-synuclein transmission and neuroinflammation. Mol. Neurodegener. 13:43. doi: 10.1186/s13024-018-0276-2, 30092810 PMC6085656

[ref36] Kim J. S. Sung H. Y. (2015). Gastrointestinal autonomic dysfunction in patients with Parkinson's disease. J Mov Disord 8, 76–82. doi: 10.14802/jmd.15008, 26090079 PMC4460543

[ref37] Lee H. J. Khoshaghideh F. Patel S. Lee S. J. (2004). Clearance of alpha-synuclein oligomeric intermediates via the lysosomal degradation pathway. J. Neurosci. 24, 1888–1896. doi: 10.1523/JNEUROSCI.3809-03.2004, 14985429 PMC6730405

[ref38] Lee P. H. Lee G. Park H. J. Bang O. Y. Joo I. S. Huh K. (2006). The plasma alpha-synuclein levels in patients with Parkinson's disease and multiple system atrophy. J. Neural Transm. (Vienna) 113, 1435–1439. doi: 10.1007/s00702-005-0427-9, 16465458

[ref39] Lee H. J. Patel S. Lee S. J. (2005). Intravesicular localization and exocytosis of alpha-synuclein and its aggregates. J. Neurosci. 25, 6016–6024. doi: 10.1523/JNEUROSCI.0692-05.2005, 15976091 PMC6724798

[ref40] Lee H. J. Suk J. E. Patrick C. Bae E. J. Cho J. H. Rho S. . (2010). Direct transfer of alpha-synuclein from neuron to astroglia causes inflammatory responses in synucleinopathies. J. Biol. Chem. 285, 9262–9272. doi: 10.1074/jbc.M109.081125, 20071342 PMC2838344

[ref41] Levitt M. D. (1969). Production and excretion of hydrogen gas in man. N. Engl. J. Med. 281, 122–127. doi: 10.1056/NEJM196907172810303, 5790483

[ref42] Li Z. Liang H. Hu Y. Lu L. Zheng C. Fan Y. . (2023). Gut bacterial profiles in Parkinson's disease: a systematic review. CNS Neurosci. Ther. 29, 140–157. doi: 10.1111/cns.13990, 36284437 PMC9804059

[ref43] Liddle R. A. (2018). Parkinson's disease from the gut. Brain Res. 1693, 201–206. doi: 10.1016/j.brainres.2018.01.010, 29360467 PMC6003841

[ref44] Liu M. Bing G. (2011). Lipopolysaccharide animal models for Parkinson's disease. Parkinson's Dis. 2011:327089. doi: 10.4061/2011/327089, 21603177 PMC3096023

[ref45] Menozzi E. Macnaughtan J. Schapira A. H. V. (2021). The gut-brain axis and Parkinson disease: clinical and pathogenetic relevance. Ann. Med. 53, 611–625. doi: 10.1080/07853890.2021.1890330, 33860738 PMC8078923

[ref46] Mou Y. Du Y. Zhou L. Yue J. Hu X. Liu Y. . (2022). Gut microbiota interact with the brain through systemic chronic inflammation: implications on Neuroinflammation, neurodegeneration, and aging. Front. Immunol. 13:796288. doi: 10.3389/fimmu.2022.796288, 35464431 PMC9021448

[ref47] Murros K. E. (2022). Hydrogen sulfide produced by gut Bacteria may induce Parkinson's disease. Cells 11. doi: 10.3390/cells11060978, 35326429 PMC8946538

[ref48] Murros K. E. Huynh V. A. Takala T. M. Saris P. E. J. (2021). *Desulfovibrio* bacteria are associated with Parkinson's disease. Front. Cell. Infect. Microbiol. 11:652617. doi: 10.3389/fcimb.2021.652617, 34012926 PMC8126658

[ref49] Nakashima A. Hayashi N. Kaneko Y. S. Mori K. Sabban E. L. Nagatsu T. . (2009). Role of N-terminus of tyrosine hydroxylase in the biosynthesis of catecholamines. J. Neural Transm. (Vienna) 116, 1355–1362. doi: 10.1007/s00702-009-0227-8, 19396395

[ref50] Nie S. Jing Z. Wang J. Deng Y. Zhang Y. Ye Z. . (2023). The link between increased Desulfovibrio and disease severity in Parkinson's disease. Appl. Microbiol. Biotechnol. 107, 3033–3045. doi: 10.1007/s00253-023-12489-1, 36995383

[ref51] Perez R. G. Waymire J. C. Lin E. Liu J. J. Guo F. Zigmond M. J. (2002). A role for alpha-synuclein in the regulation of dopamine biosynthesis. J. Neurosci. 22, 3090–3099. doi: 10.1523/JNEUROSCI.22-08-03090.2002, 11943812 PMC6757524

[ref52] Pisa D. Alonso R. Carrasco L. (2020). Parkinson's disease: a comprehensive analysis of Fungi and Bacteria in brain tissue. Int. J. Biol. Sci. 16, 1135–1152. doi: 10.7150/ijbs.42257, 32174790 PMC7053320

[ref53] Polymeropoulos M. H. Lavedan C. Leroy E. Ide S. E. Dehejia A. Dutra A. . (1997). Mutation in the alpha-synuclein gene identified in families with Parkinson's disease. Science 276, 2045–2047. doi: 10.1126/science.276.5321.2045, 9197268

[ref54] Rausch W. D. Wang F. Radad K. (2022). From the tyrosine hydroxylase hypothesis of Parkinson's disease to modern strategies: a short historical overview. J. Neural Transm. (Vienna) 129, 487–495. doi: 10.1007/s00702-022-02488-3, 35460433 PMC9188506

[ref55] Ritz N. L. Burnett B. J. Setty P. Reinhart K. M. Wilson M. R. Alcock J. . (2016). Sulfate-reducing bacteria impairs working memory in mice. Physiol. Behav. 157, 281–287. doi: 10.1016/j.physbeh.2016.01.023, 26861176

[ref56] Ritz N. L. Lin D. M. Wilson M. R. Barton L. L. Lin H. C. (2017). Sulfate-reducing bacteria slow intestinal transit in a bismuth-reversible fashion in mice. Neurogastroenterol. Motil. 29. doi: 10.1111/nmo.12907, 27477318

[ref57] Ryman S. Vakhtin A. A. Richardson S. P. Lin H. C. (2023). Microbiome-gut-brain dysfunction in prodromal and symptomatic Lewy body diseases. J. Neurol. 270, 746–758. doi: 10.1007/s00415-022-11461-9, 36355185 PMC9886597

[ref58] Salvatore M. F. Pruett B. S. Dempsey C. Fields V. (2012). Comprehensive profiling of dopamine regulation in substantia nigra and ventral tegmental area. J. Vis. Exp. 10:4171. doi: 10.3791/4171, 22907542 PMC3487291

[ref59] Sampson T. R. Challis C. Jain N. Moiseyenko A. Ladinsky M. S. Shastri G. G. . (2020). A gut bacterial amyloid promotes alpha-synuclein aggregation and motor impairment in mice. eLife 9:e53111. doi: 10.7554/eLife.53111 32043464 PMC7012599

[ref60] Scheperjans F. Aho V. Pereira P. A. Koskinen K. Paulin L. Pekkonen E. . (2015). Gut microbiota are related to Parkinson's disease and clinical phenotype. Mov. Disord. 30, 350–358. doi: 10.1002/mds.26069, 25476529

[ref61] Shannon K. M. Keshavarzian A. Mutlu E. Dodiya H. B. Daian D. Jaglin J. A. . (2012). Alpha-synuclein in colonic submucosa in early untreated Parkinson's disease. Mov. Disord. 27, 709–715. doi: 10.1002/mds.23838, 21766334

[ref62] Singh S. B. Braun C. A. Carroll-Portillo A. Coffman C. N. Lin H. C. (2024). Sulfate-reducing bacteria induce pro-inflammatory TNF-alpha and iNOS via PI3K/Akt pathway in a TLR 2-dependent manner. Microorganisms 12:1833. doi: 10.3390/microorganisms12091833 39338507 PMC11434237

[ref63] Singh S. B. Carroll-Portillo A. Lin H. C. (2023). Desulfovibrio in the gut: the enemy within? Microorganisms 11:1772. doi: 10.3390/microorganisms11071772, 37512944 PMC10383351

[ref64] Singh S. B. Coffman C. N. Carroll-Portillo A. Varga M. G. Lin H. C. (2021). Notch signaling pathway is activated by sulfate reducing Bacteria. Front. Cell. Infect. Microbiol. 11:695299. doi: 10.3389/fcimb.2021.695299, 34336718 PMC8319767

[ref65] Singh S. B. Coffman C. N. Varga M. G. Carroll-Portillo A. Braun C. A. Lin H. C. (2022). Intestinal alkaline phosphatase prevents sulfate reducing Bacteria-induced increased tight junction permeability by inhibiting snail pathway. Front. Cell. Infect. Microbiol. 12:882498. doi: 10.3389/fcimb.2022.882498, 35694541 PMC9177943

[ref66] Spillantini M. G. Schmidt M. L. Lee V. M. Trojanowski J. Q. Jakes R. Goedert M. (1997). Alpha-synuclein in Lewy bodies. Nature 388, 839–840. doi: 10.1038/42166, 9278044

[ref67] Svensson E. Horvath-Puho E. Thomsen R. W. Djurhuus J. C. Pedersen L. Borghammer P. . (2015). Vagotomy and subsequent risk of Parkinson's disease. Ann. Neurol. 78, 522–529. doi: 10.1002/ana.24448, 26031848

[ref68] Thapa M. Kumari A. Chin C. Y. Choby J. E. Jin F. Bogati B. . (2023). Translocation of gut commensal bacteria to the brain. bioRxiv. doi: 10.1101/2023.08.30.555630, 37693595 PMC10491268

[ref69] Tsigelny I. F. Crews L. Desplats P. Shaked G. M. Sharikov Y. Mizuno H. . (2008). Mechanisms of hybrid oligomer formation in the pathogenesis of combined Alzheimer's and Parkinson's diseases. PLoS One 3:e3135. doi: 10.1371/journal.pone.0003135, 18769546 PMC2519786

[ref70] Yu S. Zuo X. Li Y. Zhang C. Zhou M. Zhang Y. A. . (2004). Inhibition of tyrosine hydroxylase expression in alpha-synuclein-transfected dopaminergic neuronal cells. Neurosci. Lett. 367, 34–39. doi: 10.1016/j.neulet.2004.05.118, 15308292

[ref71] Zhang X. Tang B. Guo J. (2023). Parkinson's disease and gut microbiota: from clinical to mechanistic and therapeutic studies. Trans. Neurodegener. 12:59. doi: 10.1186/s40035-023-00392-8, 38098067 PMC10722742

[ref72] Zhang F. Yue L. Fang X. Wang G. Li C. Sun X. . (2020). Altered gut microbiota in Parkinson's disease patients/healthy spouses and its association with clinical features. Parkinsonism Relat. Disord. 81, 84–88. doi: 10.1016/j.parkreldis.2020.10.034, 33099131

