# Supplementary material for: Sulfate reducing bacteria induce α-synuclein in intestinal and neuronal cells and tissues and inhibit tyrosine hydroxylase in neuronal cells
Source: Front Neurosci. 2025 Dec 18;19:1672793. doi: 10.3389/fnins.2025.1672793 (PMC12756163; doi:10.3389/fnins.2025.1672793)

**Fig.S1. *B.thetaiotaomicron* (B.theta) does not affect synuclein aggregation in or its release from STC-1** B.theta (ATCC 29148) was grown anaerobically in chopped meat medium (Anaerobe Systems) for 24 hr in hungate tubes in a 5-ml culture. STC-1 cells were infected with either DSV or B.theta (MOI 80) for 24 h. Cells were fixed and processed for immunofluorescence using anti- α-syn aggregate antibody and the filtered culture sup were analyzed for the presence of extracellular α-syn by ELISA according to manufacturer’s protocol. **A.** Percentage of cells positive for α-syn aggregates were counted and values were compared to control (uninfected) cells. **B.** Fold change increase in a-syn in culture sup relative to control, by ELISA. Data represent mean±SEM. Data was analyzed using students t-test to compare DSV and B.theta values with control **P<0.05, ****P<.0001

**A B**


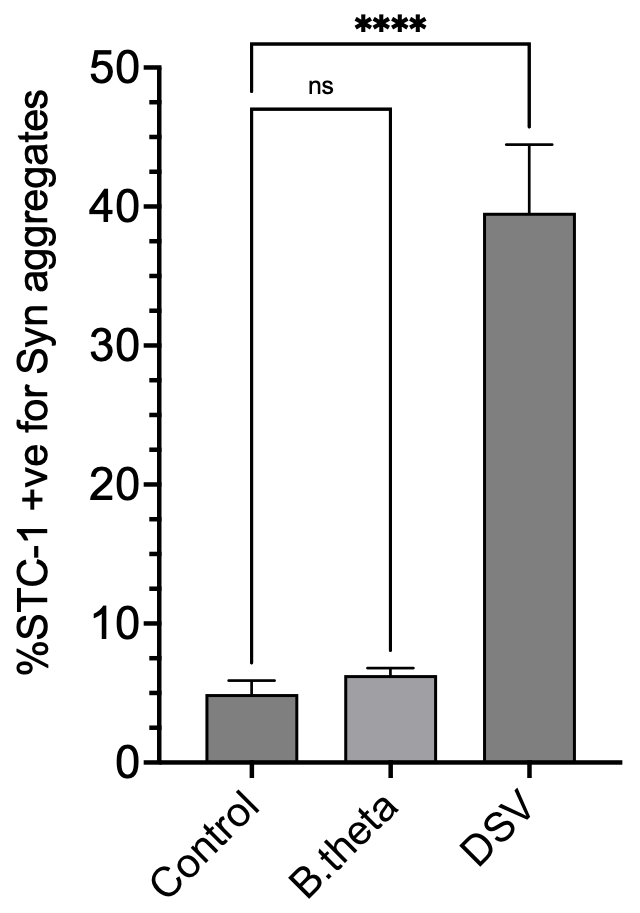

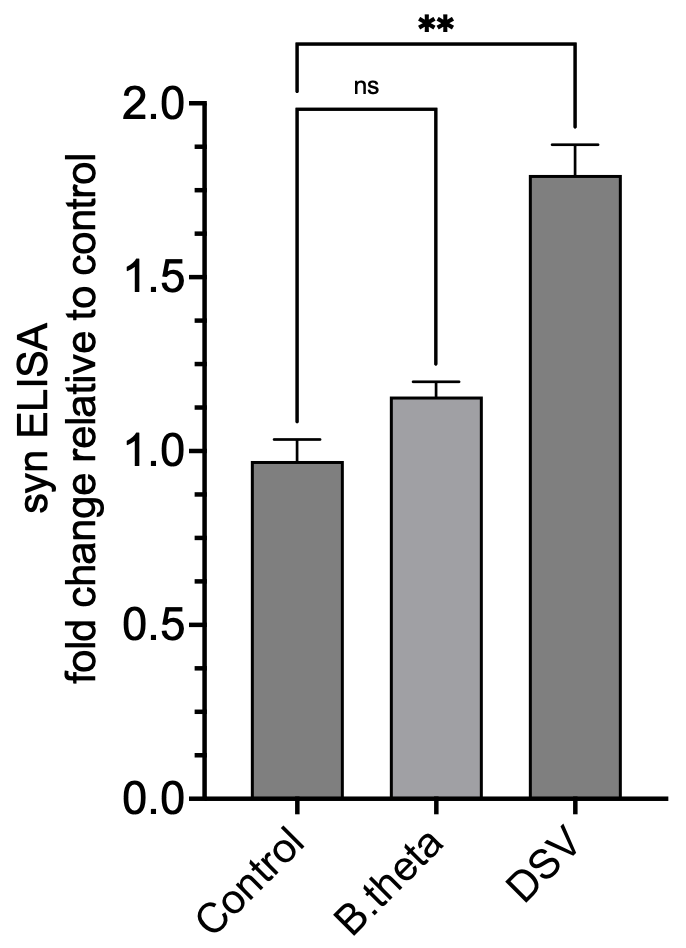

Supplement: Supplementary file 1 [file Data_Sheet_1.docx]
